# Right to Occupational Safety: Prevalence of Latent Tuberculosis Infection in Healthcare Workers. A 1-Year Retrospective Survey Carried out at Hospital of Lecce (Italy)

**DOI:** 10.3390/epidemiologia4040038

**Published:** 2023-10-31

**Authors:** Gabriele d’Ettorre, Stela Karaj, Prisco Piscitelli, Osvaldo Maiorano, Carmen Attanasi, Roberta Tornese, Eugenia Carluccio, Paolo Giannuzzi, Enrico Greco, Giancarlo Ceccarelli, Gabriella d’Ettorre, Giambattista Lobreglio, Pierpaolo Congedo, Francesco Broccolo, Alessandro Miani

**Affiliations:** 1 Vito Fazzi Hospital, Local Health Authority ASL Le, 73100 Lecce, Italy; medicomp@asl.lecce.it (G.d.); osvaldomaiorano@gmail.com (O.M.); carmen.attanasi58@gmail.com (C.A.); roberta.tornese@libero.it (R.T.); eugeniacarlu@gmail.com (E.C.); paolo.giannuzzi@asl.lecce.it (P.G.); giambattista.lobreglio@asl.lecce.it (G.L.); pierpaolo.congedo@uniroma.it (P.C.); 2 Faculty of Social Sciences, European University of Tirana, 1000 Tirana, Albania; stela.karaj@uet.edu.al; 3 Department of Experimental Medicine, University of Salento, 73100 Lecce, Italy; broccolof@gmail.com; 4 Italian Society of Environmental Medicine, 20123 Milan, Italy; enrico.greco@units.it (E.G.); alessandro.miani@gmail.com (A.M.); 5 Department of Public Health and Infectious Diseases, Sapienza University of Rome, 00185 Rome, Italy; giancarlo.ceccarelli@uniroma1.it (G.C.); gabriella.dettorre@uniroma1.it (G.d.)

**Keywords:** tuberculosis, LTBI, healthcare workers, Mantoux tuberculin skin test (TST), interferon gamma release assay (IGRA), prevention

## Abstract

Background: Prevention of latent tuberculosis infection (LTBI) in healthcare workers (HCWs) to ensure the “Right to Occupational Safety” is a special challenge globally, as HCWs have a higher risk of acquiring the infection in hospital settings because of frequent close exposure to patients suffering from tuberculosis (TB). Methods: Aretrospective study was performed with the aim of assessing the prevalence of LTBI related to demographical and occupational risk factors among HCWs employed in a large hospital in Italy. The study involved 1461 HCWs screened for LTBI by Mantoux tuberculin skin test (TST) and then confirmed with Interferon Gamma Release Assay (IGRA) test in case of positivity. Immunosuppressed and BGC-vaccinated workers were tested directly with IGRA. Results: LTBI was diagnosed in 4.1% of the HCWs and the prevalence resulted lower than other studies conducted in low TB incidence countries. The variables significantly linked with higher frequency of the infection were: age ≥40 years (OR = 3.14; 95% CI: 1.13–8.74; *p* < 0.05), length of service ≥15 years (OR = 4.11; 95% CI: 1.48–11.43; *p* < 0.05) and not being trained on TB prevention (OR = 3.46; 95% CI: 1.85–6.46; *p* < 0.05). Not trained HCWs presented a higher risk of LTBI also after adjustment for age and length of service, compared to trained HCWs. Conclusions: screening of HCWs for LTBI should be always considered in routinely occupational surveillance in order to early diagnose the infection and prevent its progression. Safety policies in hospital settings centered on workers’ training on TB prevention is crucial to minimize LTBI occurrence in HCWs.

## 1. Introduction

Prevention of tuberculosis (TB) among healthcare workers (HCWs) employed in hospital settings and exposed to occupational risk of acquiring TB is a special challenge for both healthcare organizations and HCWs worldwide in order to ensure the “Right to occupational safety”. According to the World Health Organization (WHO), TB is one of the ten major causes of death resulting from a single infectious agent, ranking above HIV/AIDS. A growing literature reveals that HCWs are exposed to the risk of occupational TB acquired in hospital setting due to the frequency of exposure to TB through droplet nuclei aerosilized by patients suffering from infectious pulmonary TB, regardless of economic setting and local TB incidence [1,2,3,4,5,6,7]. In the past, a meta-analysis performed by Baussano et al. [8] showed that HCWs present a greater risk of TB compared to general population; in fact, the median annual incidence of TB among HCWs was 67 cases/100,000 (IQR 40–142), 91/100,000 (IQR 81–723), and 1180/100,000 (IQR 91–3222) in countries with low, intermediate, and high TB incidence, respectively. The median incidence of TB detected in the general population was 33/100,000 (IQR 27–37), 82/100,000 (IQR 58–223), and 311/100,000 (IQR 168–405), respectively. Latent tuberculosis infection (LTBI) is a state of immune response to stimulation by Mycobacterium tuberculosis antigens without evidence of clinically manifested active TB, so that the preventive treatment of subjects with LTBI is recommended to eliminate the disease [9,10].

It is estimated that 23% of world’s population and 13.7 of European population have LTBI; in Italy, the incidence rate of TB in the general population is 7.2/100,000 (according to the Italian Institute of Health), but the prevalence of LTBI among the general population has not been established yet [11,12]. To date, there is a lack of information also about LTBI prevalence among HCWs, because of the poor number of studies focused on HCWs and the different diagnostic methods adopted for the diagnosis in a population which was frequently vaccinated with BCG (Calmette-Guerin Bacillus) in the past [13].

Given the risk of TB transmission in healthcare settings, LTBI screening should be prioritized for HCWs, with the aim to early detect and treat the infection and consequently prevent TB reactivation.

HCWs may be infected with Mycobacterium tuberculosis in hospital settings from infected patients or become infected in the community settings, thus representing themselves a source of infection for their colleagues and patients (in case they get infected with contagious tuberculosis).

The WHO strategy and framework towards tuberculosis elimination in low-incidence countries stated that LTBI screening and treatment in high-risk groups are priority actions to reach the goal of controlling and eliminating TB [14,15]. Most people infected with M. tuberculosis have no signs or symptoms of TB and will never develop the disease. The risk of developing active tuberculosis following infection is related to age, the quality of immune defense mechanisms and the time elapsed since infection. According to recent data, the estimated risk is 5–10%, but it is higher in young children, immunocompromised individuals, and within the first 1–2 years after the contact with a contagious TB case [13].

The European Centers for Disease Control (ECDC) underlined the need for high-quality management program for LTBI and provided a specific program targeted on groups at higher risk for LTBI or presenting a risk of progressing to active TB [16]. In line with ECDC statement, periodic TB testing of HCWs is recommended as part of TB infection control plan and Mantoux tubercolin skin test (TST) represents the standard method for detecting LTBI in HCWs [17]. Nevertheless, the low TST specificity among BCG-vaccinated HCWs and the boosting phenomenon of repeated TST may provide false positive results, with a potential negative consequence of unnecessary chest X-rays prescriptions and/or medications for LTBI [18]. The interferon gamma release assay (IGRA) is a specific and rapid alternative to the TST.

The IGRA requires only one patient visit and is unaffected by previous BCG vaccination or nontuberculous mycobacteria infections.

However, given the high cost of IGRA, the TST remains the suggested method for LTBI diagnosis, at least in Italy. Therefore, IGRA is recommended only in immunosuppressed patients, in HCWs vaccinated with BCG vaccine, in children <5 years and in order to confirm the positivity to TST tests. Regarding anti-TB vaccine, the only one existing since 1921 is a live attenuated vaccine (BCG), which protects for 5–10 years but is not effective in the following period neither for the prevention of the disease nor for the interruption of TB transmission in the population. Actually, in Italy anti-TB vaccination is not compulsory for everyone in healthcare settings [17].

The present study was aimed at assessing the prevalence of LTBI related to demographical and occupational risk factors among HCWs employed in a large Hospital such as Vito Fazzi Hospital in Lecce (Salento), Italy.

## 2. Methods

A retrospective survey was performed by analyzing the occupational medicine database of HCWs employed in a large hospital (Vito Fazzi Hospital, Lecce) located in Salento (the Southern part of Puglia region, in Italy), who underwent the routine annual occupational health surveillance from 1 November 2021 to 31 December 2022. The sample included medical doctors, nurses, laboratory and radiology technicians. In order to evaluate the prevalence of LTBI cases, the results of Mantoux TSTs were analyzed.

All the population of HCWs who underwent health surveillance was tested for TB infection. In our study, the HCWs were not previously tested for TB infection. Mantoux tuberculin skin test was performed in HCWs by intradermal injection of 0.1 mL (2 units) of tuberculin PPD into the dorsal surface of the forearm and if positive was done the IGRA. The TST result was ready after 72 h by measuring the diameter of induration, transversely to long axis of the forearm using a standardized ruler. Mantoux tuberculin skin test was considered positive in case of diameter of induration of at least 10 mm. According to Italian National Anti- Tuberculosis Program, the TST induration ≥10 mm is considered positive. Nevertheless, other studies and guidelines developed by Centres for Disease Control (CDC) and the American Thoracic Society (ATS) assume that for individuals who are not in high risk groups, LTBI can be diagnosed if TST induration is ≥15 mm [17,19].

With regard to the occupational risk assessment of TB infection, in 2013 the Italian Ministry of Health has classified occupational settings into increasing levels of risk (low-medium-high) associated to growing risk of infection [10]. For this research the author used the Occupational Prevention and Protection Service database of the hospital, consisting of all clinical reports and personal information, institutionally maintained at hospital level in the frame of current job policies. The study was performed as part of the mandatory assessment of occupational LTBI, required by the Italian Legislative Decree 81/2008 and therefore needed no formal approval by the local ethics committee.

### Statistical Analyses

Data were analyzed with the SPSS software package (Statistical Package for Social Sciences), version 14.0. Analysis of the frequency of individual variables was conducted using descriptive statistics. Univariate analysis included the Student *t*-test for quantitative and the chi-square test for categorical variables. Comparisons between groups were performed with the Mann-Whitney U test for nonparametric data in the case of two independent groups. The statistical significance was set at *p* < 0.05 for all analyses. The statistical analysis included a logistic regression analysis to calculate the odd ratio (OR) with 95% confidence interval. In this study, the independent variables were: age, length of service, job task, training on TB prevention, reported exposure to active TB, being BCG vaccinated, occupational risk for TB infection, and the dependent variable was LTBI.

## 3. Results

The study involved 1421 HCWs (446 males and 975 females), mean age 49.3 (±10.0) years old; demographics data are shown in Table 1. Diagnosis of LTBI was posed for 58 HCWs (4.1%) with no previous positivity. Age ≥ 40 years (OR = 3.14; 95% CI= 1.13–8.74 *p* < 0.05) length of service ≥ 15 years (OR = 4.11; 95% CI= 1.48–11.43 *p* < 0.05) and not being trained on TB prevention (OR = 3.46; 95% CI= 1.85–6.46 *p* < 0.05) were the variables significantly linked with higher frequency of LTBI in the study population (Table 2). With regard to training on TB prevention, the non-trained HCWs resulted at higher risk of LTBI also after stratification for age and length of service (Table 3). No significant differences were found among the HCWs compared by gender, job task, exposure to high risk of occupational TB, being BCG vaccinated and reporting exposure to active TB in the past (Table 2). All the HCWs who were positive at TST resulted positive at IGRA test; no case of active TB was diagnosed among the HCWs included in this study.

## 4. Discussion

According to the WHO action plan for TB elimination in low-incidence countries, an occupational LTBI control program for HCWs based on workers’ screening for TB was adopted. The safety policies taken by the Administration and the Occupational Medicine facility of the investigated hospital were proved to be effective and allowed to achieve the goal of minimizing the occupational TB risk. Actually, healthcare settings are regarded as being at high risk of contact with TB infectious patients, so that early diagnosis of LTBI allows for timely treatment and, consequently, minimizes the risk of progression from LTBI to active TB [14,15].

With regard to TST, the test is characterized by a large number of drawbacks that make its use less than ideal. Moreover, tuberculin solution contains a large number of antigens (~200), most of which are common to *M. tuberculosis*, *M. bovis*, *BCG*, and environmental mycobacteria: therefore, a positive test reaction can be elicited by a prior vaccination with BCG or a contact with environmental mycobacteria and presents a low specificity. Furthermore, the technique for placing tuberculin and reading the reaction can be associated with several technical errors (e.g., injection in the wrong skin layer, fluid leaks, subjective evaluation of the skin reaction with intra- and inter-observer errors). Several conditions can decrease the skin reactivity, such as viral infections, immune depression, young and old age. Repeating the test can elicit an artificial increase in size of the second reaction (the so-called “booster effect”) [13].

Healthcare workers who were found positive at TST underwent IGRA test for confirmation of positivity and were studied by specific clinical examination and chest X-ray to exclude the presence of active TB.

Both TST and IGRA are based on immune response of activated antigen presenting cells and sensitized lymphocytes to *M. tuberculosis* antigens. The TST relies on the reaction to intradermal injection of tuberculin, which induces a local inflammatory induration that can be measured, whereas IGRAs are in vitro tests measuring IFN-γ released from CD4+ lymphocytes upon stimulation by ESAT-6 and CFP-10 (two antigens encoded in the region of difference 1 locus present in *M. tuberculosis* genome and also present in tuberculin).

Given that TST could be affected by false negativity in immunosuppression conditions, HCWs immunosuppressed were screened with IGRA test and not with TST. Healthcare workers who were BCG-vaccinated underwent IGRA test and not TST because of the low specificity of TST in such workers. Interferon gamma release assay results are reported results are reported as one of the following: positive, negative, indeterminate or uninterpretable results. According to the manufacturer’s published criteria, the test was classified as positive when the antigen specific IFN gamma serum value was equal or higher than the cut-off level of 0.35 IU/mL, compared to a negative control [20]. Latent tuberculosis infection diagnosis was posed in HCWs with positive IGRA test and not suffering from active TB. Healthcare workers with a diagnosis of LTBI were examined by an infectious diseases specialist and were enrolled for a clinical follow up after diagnosis. An infectious diseases consultant evaluated all LTBI cases and the decision to prescribe medications to treat LTBI was made on a case-by-case basis.

In our study the prevalence of LTBI among HCWs was lower compared to data of other studies performed in healthcare settings in low TB incidence countries [1,13,18] and consistent with the findings of Coppeta et al. [10,12]. HCWs aged ≥ 40 years were found at higher risk of LTBI; consistently with this finding and with the review performed by da Silva et al. [21], an increased OR for LTBI was found in HCWs with length of service ≥ 15 years compared to low-experienced HCWs. These findings highlighted the significant relationship between years of exposure to occupational TB risk in hospital settings and LTBI. This finding was similar to that of Park JS [22], that conducted a study among HCWs demonstrating that both age (OR = 1.094; 95% CI, 1.064–1.124) and duration of work (OR = 1.094; 95% CI, 1.096; 95% CI, 1.065–1.128) were independent variables related to significant risk of LTBI.

Moreover, the study performed by Szturmowicz et al. [19] revealed that the risk of obtaining positive IGRA in medical workers was increased in those above 44 years of age (OR = 4,92; 95% IC 2.4–10.2) and employed from more than 10 years (OR = 2,73; 95% IC 1.1–6.6).

Interestingly, our study provides evidence of the protective role of TB prevention training in health care settings: prevalence of LTBI was higher in untrained HCWs compared to trained personnel (OR = 3.46; 95% IC 1.85–6.46); the protective role of training was confirmed also after correction for age and length of service. This finding underlines the strategic role of occupational training targeted at occupational risks as a way to minimize the LTBI risk in HCWs by increasing the workers’ risk perception and by promoting the adoption of safe behaviors. In fact, prior studies proved a relationship between risk perception and protective behaviors in workers exposed to occupational hazards; in particular, Savadori et al. [23] revealed that risk perceptions were antecedents of protective behaviors and suggested that the effective communication of risks could represent a strategy to improve health-protective behaviors among HCWs.

According to our findings, specific training should improve knowledge and skills so that HCWs can identify TB hazards, change attitudes, and adopt safe work behaviors to avoid occupational exposure to these hazards. These targets recognize the potential of occupational training as a promising way to help HCWs minimizing occupational hazards in healthcare settings. An empowerment perspective prioritizes workers’ role in removing or controlling hazards. To achieve this goal, health and safety training must be designed and conducted in ways that teach HCWs how to identify hazards as well as how to deal effectively with collective action to move employers, the healthcare organization, and even employees to remove or maximally control workplace hazards. By doing so, workers can take effective actions to end working conditions and exposures that rise their risk of contracting LTBI due to their work.

Occupational health practice prioritizes removing hazards entirely as the best solution to prevent HCWs from suffering dangerous exposures in the healthcare settings. Nevertheless, when occupational hazards cannot be removed, controlling them to maximally prevent exposures is the most effective approach and worker training is a way for both minimizing hazardous exposures and for bringing workers’ knowledge of work processes into the decision-making process about establishing healthy and safe workplaces. In day-to-day experience, this is often done when activities require the use of personal protective equipment (PPE), to help improve the protection potential of the PPE being used. In this context, training is an important vehicle for improving workers’ understanding of workplace hazards and the health and safety risks they pose.

The Italian legislative Decree 81/2008 concerning the protection of health and safety at workplaces highlighted the strategic role of workers’ training on biological hazards in order to protect safety and health of employees. Occupational health professionals are asked to address issues related to prevention of LTBI in healthcare settings by ensuring workers’ training focused on TB hazard at workplaces. In this context, the occupational medicine physicians, while carrying out their activity, must be able to interact-with increasing and multidisciplinary competences-with other professionals involved in the risk prevention system such as employers, employees, safety and industrial hygiene professionals, occupational health and safety services of the companies, personnel representative bodies and labor unions, general practitioners and public health care professionals. Moreover, occupational medicine physicians are increasingly asked to address issues of environmental medicine and health promotion in addition to protecting the worker’s safety and health against work-related injuries and occupational diseases. Therefore, there is an evident need to determine and periodically reevaluate professional activity (and the related skills and competencies) and the information demands and/or education and training needs of HCWs, in order to ensure their adequate protection and continually improve their health and safety in hospital settings.

With regard to primary prevention of LTBI through vaccination, no evidence was found about the protective role of BCG vaccination, given that no significant differences were found among HCWs BCG vaccinated compared to unvaccinated. Our finding is in agreement with other studies [10,12,24] conducted among HCWs working in countries with low incidence of TB.

To date, in Italy anti-TB vaccination is not compulsory for everyone and in 2013, the Italian Ministry of Health launched the latest TB prevention national guidelines for HCWs, indicating TST as the first-level screening test [25]. When TST is positive, results have to be confirmed by enzyme immunoassay tests based on IGRA quantification, proving a superior specificity and sensitivity if compared with TST [26]. In particular, IGRA in HCWs has excellent utility and accuracy, especially in BCG-vaccinated populations in low incidence countries [27,28,29], but the test is greatly expensive.

Finally, in the present study we assessed the correlation between the TB risk in the areas of employment and the prevalence of LTBI among HCWs: the analysis showed that working in high risk setting was not related to a greater prevalence of LTBI (OR = 1.01; 95% IC 0.60–2.03). This finding is in line with the study of Coppeta et al. [10] and provides evidence about the need of serial screening for LTBI involving all HCWs in hospital settings, regardless to risk classification of the employment setting (high, medium, low).

This study suffers from some limitations; firstly, the findings are subjected to the limitation inherent in cross-sectional data and, therefore, no causal relationship between variables can be drawn. Therefore, in future studies, causal relationships among variables should be analyzed using longitudinal study design.

Secondly, the findings could have been influenced by organizational factors intrinsic to the Italian occupational context and, consequently, not generalizable to all the healthcare settings.

Third the results presented should be referred only to the specific population analyzed in this study: all enrolled HCWs were recruited between subjects who underwent the routine annual occupational health surveillance.

Finally, the sample size is not representative of all Italian HCWs.

A strenght of the study is that all the enrolled HCWs underwent LTBI screening; HCWs who were found positive at TST underwent IGRA test for confirmation of positivity and were studied by specific clinical examination and chest X-ray to exclude the presence of active TB. Moreover, an infectious diseases consultant evaluated all LTBI cases and the decision to prescribe medications to treat LTBI was made on a case-by-case basis.

## 5. Conclusions

In our study the screening of HCWs for TB showed a lower prevalence of LTBI than other studies conducted in low TB incidence countries.

This finding proved the effectiveness of occupational safety interventions aimed at minimizing the risk of TB transmission from patients to HCWs in hospital settings; occupational training on TB prevention appears strategic to achieve the goal of minimizing LTBI risk among HCWs.

Healthcare organizations should provide occupational training focused on the TB hazard in hospital settings with the aim to improve both the workers’ awareness on occupational TB and the prevention measures targeted at controlling the risk.

It is also important to highlight that the findings of our study corroborate the evidence of low LTBI prevalence among HCWs working in Italian hospital settings, showed by recent studies [10,12].

Moreover, our study suggests the need of further LTBI surveillance in this group, regardless of working in high-risk hospital settings, and the implementation of evidence-based infection control practices. Therefore, LTBI screening should be strongly advised in medical staff, especially in those exceeding 40 years of age, or those employed for a long time with the direct contact with infective materials or patients. IGRA can be recommended for serial testing as boosting effect does not occur, contrary to repetitive tuberculin administration. Serial screening for latent TB infection should include all HCWs, regardless to risk classification of the employment setting.

### Applications to Professional Practice

Tuberculosis elimination in low-incidence countries represents a challenge for healthcare organizations all over the world. In accordance with the World Health Organization’s End TB Strategy, LTBI screening programs need to be prioritized for HCWs, given that they are at high risk of TB infection. Although LTBI is not a contagious state, the reactivation of the infection can lead to active TB, following immunosuppressive treatment or other medical conditions. In our study we investigated the prevalence of LTBI among HCWs employed in hospital setting, in Italy, a low incidence country for TB, and analyzed the variables related with LTBI occurrence. In accordance with other studies, we found that age and length of service were related with the risk of LTBI. Interestingly, we found that training on TB prevention was an independent variable related to higher risk of LTBI in HCWs. Our study provides suggestions about the strength of using participatory training approaches to help workers learn, as well as the importance of involving workers in developing the training program targeted on healthcare settings.

Occupational medicine facilities can play a key role in minimizing the TB risk among HCWs through LTBI surveillance, early diagnosis of TB and workers’ training on TB risk.

In this regard, core competencies and skills of occupational health professionals are required in order to provide methodologies and tools that may contribute to improvement of the HCWs professional knowledge and competency (e.g., introducing evidence-based medicine and professional certification processes), to address learning outcomes for effective specialist training, to influence the organization and structure of service delivery, and then, finally, to inform policy decisions.

Nowadays TB is still a major public health problem, for this reason a combined strategy, based on improving diagnostic instruments and prevention strategy of LTBI among HCWs in hospital settings, is necessary, in order to eradicate *M. Tuberculosis*, as committed by the World Health Organization [30].

## Figures and Tables

**Table 1:** Demographics of study population.

*Variables*	*N. (SD)*	*(%)*
Healthcare workers	1421	
Gender		
Females	975	68.6
Males	446	31.4
Mean age (SD)	49.3 (10.03)	
Age (years)		
<45	490	34.5
≥45	931	65.5
Lenght of service (years)		
<15	322	22.7
≥15	1099	77.3
Job		
Nurses	923	64.9
Medical doctors	298	21.0
Technical staff	189	13.3
Physiotherapists	11	0.8
Occupational risk for TB infection (*)		
High	320	22.5
Low-medium	1101	77.5
LTBI detected cases		
Positive	58	4.1
Negative	1363	95.9

(*) Tuberculosis prevention in healthcare workers. Italian Ministry of Public Health 2013.

**Table 2:** Main characteristics of latent tuberculosis infection detected cases.

*Variables*	*N. (%) of Healthcare Workers Positive for IGRA Test*	*OR (95% C.I.)*	*p Value*
**Gender**			
Females	36/975 (3.7)	1	
Males	22/446 (4.9)	1.35 (0.78–2.33)	>0.05
**Age (years)**			
<40	4/261 (1.5)	1	
≥40	54/1160 (4.6)	3.14 (1.13–8.74)	<0.05
**Lenght of service (years)**			
<15	4/322 (1.2)	1	
≥15	54/1099 (4.9)	4.11 (1.48–11.43)	<0.01
**Job**			
Nurses	34/924 (3.7)	1	
Medical doctors	13/298 (4.4)	1.19 (0.62–2.29)	>0.05
Technical staff	10/188 (5.3)	1.47 (0.71–3.03)	>0.05
Physiotherapists	1/11 (9.1)	2.61 (0.33–21.03)	>0.05
**Occupational risk for** TB infection (*)			
Low-medium	44/1101 (4.0)	1	
High	14/320 (4.4)	1.01 (0.60–2.03)	>0.05
**Training on TB** **prevention**			
Yes	13/694 (1.9)	1	
No	45/727 (6.2)	3.46 (1.85–6.46)	<0.01
**Reported exposure to** **active TB**			
Yes	5/150 (3.3)	1	
No	53/1271 (4.2)	1.26 (0.50–3.21)	>0.05
**BCG vaccinated**			
Yes	51/1165 (4.4)	1	
No	7/256 (2.7)	0.61 (0.27–1.37)	>0.05

(*) Tuberculosis prevention in healthcare workers. Italian Ministry of Public Health 2013.

**Table 3:** Latent tuberculosis infection prevalence in healthcare workers compared for age, lenght of service and training on TB prevention.

*Variables*	*Training on TB Prevention* *(N. of HCWs)*	*LTBI*		*OR*
		*Negative*	*Positive*	
**Age < 40 years**	**Yes (230)** **No (31)**	**228** **29**	2 2	8.37 (1.13–61.9)
Age ≥ 40 years	Yes (870) No (344)	851 309	19 35	5.6 (3.1–9.9)
Lenght of service (years): <15 ≥15	Yes (294) No (28) Yes (841) No (258)	292 26 821 22	2 2 20 34	12.1 (1.6–89.6) 7.16 (4.03–12.73)

## Data Availability

Data are available upon request to the corresponding author.

## Abbreviations

BCG	Calmette-Guerin Bacillus
IGRA	interferon gamma release assay
LTBI	latent tuberculosis infections
TB	tuberculosis
